# *Cannabis* Extraction Technologies: Impact of Research and Value Addition in Latin America

**DOI:** 10.3390/molecules28072895

**Published:** 2023-03-23

**Authors:** Ángela Suárez-Jacobo, Adrián Díaz Pacheco, Edgar Bonales-Alatorre, Gustavo Adolfo Castillo-Herrera, Jorge Alberto García-Fajardo

**Affiliations:** 1 Tecnología Alimentaria, Centro de Investigación y Asistencia en Tecnología y Diseño del Estado de Jalisco, Zapopan 45019, Mexico; 2 Unidad Profesional Interdisciplinaria de Ingeniería Campus Tlaxcala del Instituto Politécnico Nacional, Tlaxcala 90000, Mexico; 3 Centro Universitario de Investigaciones Biomédicas, Universidad de Colima, Colima 28045, Mexico; 4 Subsede Noreste, Centro de Investigación y Asistencia en Tecnología y Diseño del Estado de Jalisco, Parque de Investigación e Innovación Tecnológica, Apodaca 66628, Mexico

**Keywords:** cannabinoids, *Cannabis*, extraction procedures, value-added

## Abstract

The Cannabis genus of plants has been widely used in different cultures for various purposes. It is separated into three main species: sativa, indica, and ruderalis. In ancient practices, the plant was used as a multipurpose crop and valued for its fiber, food, and medicinal uses. Since methodologies for the extraction, processing, and identification of components have become available, medical, and food applications have been increasing, allowing potential development in the pharmaceutical and healthy functional food industries. Although the growing legalization and adoption of cannabis for the treatment of diseases are key factors pushing the growth of its market, the biggest challenge is to obtain higher-quality products in a time- and cost-effective fashion, making the process of extraction and separation an essential step. Latin American countries exhibit great knowledge of extraction technologies; nevertheless, it is still necessary to verify whether production costs are economically profitable. In addition, there has been an increase in commercial cannabis products that may or may not be allowed, with or without quality fact sheets, which can pose health risks. Hence, legalization is mandatory and urgent for the rest of Latin American countries. In this article, the phytochemical compounds (cannabinoids, terpenes, and phenolic compounds), the current status of legalization, extraction techniques, and research advances in cannabis in Latin America are reviewed.

## 1. Introduction

The *Cannabis* genus of plants has been widely used in diverse cultures for various purposes. Its origin is believed to have arisen in Central Asia and quickly spread throughout Europe and America [1]; it is separated into three main species, *sativa*, *indica*, and *ruderalis,* with different varieties or artificial crosses produced to enhance certain effects. The global *Cannabis sativa* market, including essential oils, foods, personal-care products, and medical formulations, has gained much attention over the last few years due to the favorable regulatory framework. Undoubtedly, the enormous interest in cannabis cultivation derives from the well-known pharmacological properties of cannabinoids and terpenes biosynthesized by plants [2]. The term “Cannabis” defines the dry or fresh leaves and flowers of *Cannabis sativa* or *Cannabis indica* plants.

The name “cannabinoid” has then been associated with the biological profile of the psychotropic constituent of marijuana, Δ^9^-tetrahydrocannabinol (THC), found within almost 200 known cannabinoids [3]. Another major component of Cannabis is cannabidiol (CBD), which has excellent medicinal utility. Specifically, CBD is considered for therapeutic use and does not generate psychotropic effects. In addition, to plant *Cannabis sativa*, two types of cannabinoids—synthetic cannabinoids (e.g., WIN55212-2) and endogenous cannabinoids (eCB), anandamide (ANA) and 2-arachidonoylglycerol (2-AG)—are known [4]. The active components of *Cannabis sativa* mimic the effects of endocannabinoids by activating specific cannabinoid receptors, particularly CB1 and CB2, and regulating a broad spectrum of physiological functions in which an alteration could potentially cause a variety of effects, namely, analgesic, neuroprotective, antiemetic, anticonvulsant, anti-inflammatory, and antispasmodic, and their therapeutic potential has been shown in cancer, epilepsy, sclerosis, neuropathic and chronic pain, spinal cord injury, Parkinson’s and Alzheimer’s diseases, post-traumatic stress disorder and anxiety, schizophrenia, and pulmonary disease [5]. Cannabis compounds have been used in other product developments and launched in international markets.

Among *Cannabis sativa* L. species, hemp and marijuana are plants with differing morphologies, chemical compositions, and marketed products. Usually, marijuana is cultivated with the purpose of producing THC, and its strains can be artificially manipulated to provide a high potency of this psychoactive molecule. New marijuana strains have been developed as hybrid varieties to increase specific characteristics (terpenes and CBD-specific content), differentiate the strain, or increase the drug’s effectiveness. Each marijuana strain can contain between 10 and 30% THC, 33 times more powerful than hemp. A low level of CBD is no longer characteristic of marijuana, as strains such as Dinamed are circulating on the market, with up to 14% CBD and about 0.5% THC (https://www.dinafem.org/es/dinamed-cbd/(accessed on 10 February 2023)).

Hemp is defined as the stems, seeds, and flowers whose harvest can produce oil, food, paper, fiber textiles, foodstuffs, building materials, and even topical ointments. In contrast to marijuana, hemp is rich in CBD molecules and contains at least 0.3% THC, decreasing the psychoactive effects. Therefore, it has been decriminalized in many countries to take advantage of its industrial uses. The World Anti-Doping Agency (WADA) changed its regulations by eliminating CBD from prohibited substances, thus allowing athletes to use CBD oils and derivatives without any repercussions. The U.S. Food and Drug Administration (FDA) has not approved the use of *Cannabis* as a medical treatment for different ailments [6], however isolated THC and CBD pharmaceuticals are licensed and approved [7]. Some FDA-approved drug products are Epidiolex (cannabidiol) and three synthetic cannabis-related drug products: Marinol (dronabinol), Syndros (dronabinol), and Cesamet (nabilone). Another treatment called Sativex^®^ is used (in Canada and a few European countries, including the United Kingdom, Germany, and Spain) as an adjunctive treatment for the symptomatic relief of neuropathic pain in multiple sclerosis [8].

The legal aspects related to the prohibition and criminalization of the consumption, use, and production of Cannabis have been a barrier to generating scientific and technological knowledge in Latin America. Lately, it has been changed, and several countries have legalized its medicinal and medical use. In this sense, the diversification of food and medical cannabis products has been developed, and technological processes considering their extraction, production, and formulation have been promoted. Developing high-efficiency extraction and purification techniques and optimizing their operating conditions at the pilot scale are essential for scaling up the industrial production of the main bioactive compounds in *C. sativa* [9].

Efficient, safe, and cost-effective extraction and purification technologies must be developed and optimized to improve the yield and selectivity of CBD and terpenes and ensure the purity and safety of the extract for food applications [10]. Extensive scientific research is needed to provide evidence of the benefits for health and safety in the consumption of phytocannabinoids, which, depending on the doses and purity of the components, will help to establish regulations for consumption in each country, first for the development of products with CBD and later those with THC.

It is essential to highlight that, in many Latin American countries, cannabis legalization requests are in progress as an alternative solution to illegal traffic and associated crime. Argentina, Brazil, Chile, Colombia, Ecuador, and Peru are permissive for cultivation for personal or medical use in small amounts. By 2022, all countries in Latin America had regulated the medical cannabis industry, except for Mexico, Honduras, Nicaragua, and El Salvador [11].

Different authorities have implemented four different models of legal cannabis production and supply: (1) taxed commercial supply, where licensed farms supply licensed retail outlets (followed by some US states, including Colorado, Washington state, Alaska, and Massachusetts); (2) government supply, where the government hires a limited number of farms and controls the supply through these outlets (Uruguay follows this model, along with two other models); (3) home growth permission, where there are no taxes or sales outlets (this is followed by Washington DC and Uruguay as well); (4) Social Clubs, in which groups of people can grow it in a collective and consume it without any taxes or sales outlets (this is the third model that Uruguay follows) [12]. Based on a recent market analysis, in 2019, the global cannabis market (regulated and illicit) was estimated at USD 344 billion, with 263 million consumers annually [13].

In this sense, the present document is organized with an emphasis on the technical extraction processing description of the main Cannabis components, the current impact of Cannabis research in Latin America, and finally, an overview of the economic effect of scaled-up processing and future value-added chains.

## 2. Data Collection

The information used in writing this review article comes from the analysis of relevant documents reported in trusted literature sources, such as primary data, books, and national or international journals, published from January 2000 until December 2022. The prior references were mainly cited from sources such as Scopus, Science Direct, Google Scholar, ResearchGate, Web of Science, and other published sources with the following keywords: cannabis components, cannabis extraction processing, Latin American research, health benefits, and market potential. Furthermore, data searches were also conducted using different online platforms. This review article does not have any inclusion criteria. Finally, the search was only limited to articles published in English, but information about the market and legislation was found in other languages.

## 3. Cannabis Extract: Varieties and Phytochemical Composition

The genus *Cannabis*, which is a member of the Cannabaceae family, is one of the oldest domesticated crops in the world due to its multiple applications, especially its healing properties [1]. It is an annual and dioecious plant, meaning it can be male or female. Furthermore, environmental factors, such as the appropriate photoperiod and a low temperature, can induce pollen production in female flowers, which leads to the development of feminized seeds [14].

Its evolution, taxonomic classification, and phylogenetic connections remain poorly understood. These shortcomings stem from scarce research due to legal and government restrictions, which have resulted in the high heterozygosity observed in *Cannabis* genomes today [14].

In the genus *Cannabis*, the exact number of species that have resulted from extensive hybridization and subsequent rehybridization of their original botanical descriptors is controversial. The genus classification includes three species with distinct phenotypic differences: *Cannabis sativa*, a tall and less furcate plant with long and narrow leaves, *Cannabis indica* Lam (Lamarck), a short and highly branched plant with broader leaves, and *Cannabis ruderalis*, a short plant with less branching and small and thick leaves [14].

*Cannabis sativa* is an economically important genus that provides protein, oil-rich seeds, and long and short fibers for industrial applications (building materials, textiles, or paper). A wide variety of secondary metabolites are found, such as terpenoids and cannabinoids, which are of interest to different industries [15]. The plant allows the acquisition of a large amount of lignocellulosic biomass in a brief time, which is why it is considered an abundant renewable source from which biopolymers, resistant fibers [16], and textiles can be obtained, and its use in feeding livestock has even been described [17].

Hemp powder’s antibacterial activity was also investigated against *Escherichia coli*. The hemp powder inhibited bacterial growth, and antibacterial agents were linked to the chemical composition of bast fibers (free and esterified sterols, triterpenes, β-sitosterol, and β-amyrin, with the presence of lignin, phenolic compounds, alkaloids, and cannabinoids) [18].

Despite scientific research on *Cannabis* being restricted by the Single Convention on Narcotic Drugs of 1961, nowadays, legislation in several countries allows its potential use for medical applications by using methodologies and technologies to study its composition and develop better extraction processing methods to obtain compounds for medical formulations.

### 3.1. Cannabis Phytochemical Composition 

Phytochemical components of *Cannabis sp*. are represented by cannabinoids, flavones, and terpenes, which have been the object of study in research works. Forty years ago, Turner et al. [19] reported almost 421 total compounds. More than 560 components have been identified in *Cannabis*, considered natural product phytocompounds [20]. Among these components, 120 are in the typical C21 group of compounds, known as total cannabinoids, including their analogs and transformation products. Other components include nitrogenous compounds (27), amino acids (18), proteins (3), enzymes (6), glycoproteins (2), sugars and related compounds (34), hydrocarbons (50), simple alcohols (7), simple aldehydes (12), simple ketones (13), simple acids (20), fatty acids (23), simple esters (12), lactones (1), steroids (11), terpenes (120), non-cannabinoid phenols (25), vitamins (1), pigments (2), and elements (9).

#### 3.1.1. Cannabinoids

**Figure 1:**
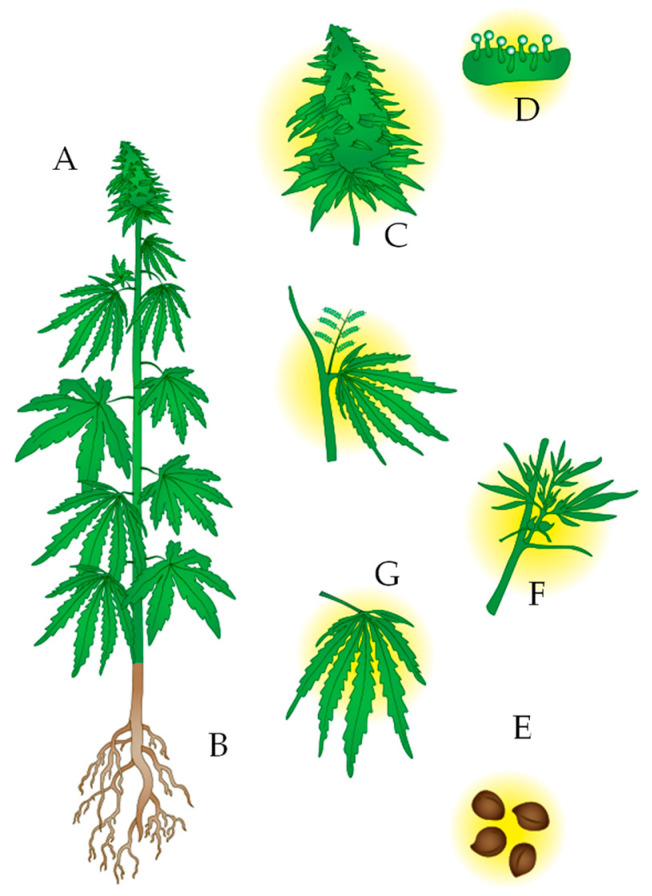
Representation of a *Cannabis sativa* plant. (**A**). The plant is usually tall and not highly branched, with long and narrow leaves. Depending on whether it is the drug type or fiber type, the stem can be thin or thick, respectively. (**B**). It has fasciculate roots. (**C**). The flower, in the case of female plants, develops in the upper part of the plant. Male plants do not have a flower. (**D**). Glandular trichomes store secondary metabolites, both cannabinoids and terpenes or phenolic compounds. (**E**). From the seeds, the oil can be extracted, and there are high contents of THC and CBD in their bark since they are in contact with the leaves of the plant. (**F**). Stem. (**G**). Leaves.

Cannabinoids represent more than 20% of secondary metabolites isolated from the cannabis plant [21]. Cannabinoids belong to the chemical class of terpene phenolics, widely distributed in nature. These metabolites are produced by, stored in, and secreted from the glandular trichomes of female flowers as a defense mechanism [22]. Trichomes are epidermal protrusions that line plants’ leaves, braces, and stems. However, their location in other parts of the plant is possible, although to a lesser extent (Figure 1), e.g., seeds, leaves, roots, and pollen [16,23]. Livingston et al. [24] concluded that revealing the particular and uncommon properties of these economically and biotechnologically important structures provides new opportunities to optimize the harvest time and extraction processing for the obtention of cannabinoids.

Cannabinoids can be produced by employing two metabolic pathways that have already been studied: the polyketide pathway, which gives rise to olivetolic acid (OLA), and the methylerythritol phosphate pathway (MEP), which leads to the synthesis of geranyl diphosphate (GPP) [14]. In 1999, Pate proposed the term phytocannabinoids for C21 compounds produced by cannabis [25]. One hundred twenty cannabinoids have been found, and the main active constituent is Δ9-tetrahydrocannabinol (Δ9 -THC), generated in the leaves and flower sprouts of the plant. *C. sativa* L. with a Δ9-THC content of less than 0.3% is cultivated as hemp, and Cannabis containing more than 0.3 percent THC is considered a medical marijuana product [26]. Cannabinoids have been classified into 11 types: (-)-Δ9-*trans*-tetrahydrocannabinol (Δ9-THC), (-)-Δ8-*trans*-tetrahydrocannabinol (Δ8-THC), cannabigerol (CBG), cannabichromene (CBC), cannabidiol (CBD), cannabinodiol (CBND), cannabielsoin (CBE), cannabicyclol (CBL), cannabinol (CBN), and cannabitriol (CBT). Some of them, such as cannabidiol (CBD), cannabichromene (CBC), cannabigerol (CBG), and cannabinol (CBN), have demonstrated non-psychoactive effects but with demonstrated pharmacological activity. The presence of terpenes and flavonoids in Cannabis extracts improves the biological activities of the cannabinoids [27].

THC is the principal psychoactive substance and is abundant in *Cannabis* drug-type plants. It induces feelings of euphoria, anxiety, paranoia, and cognitive deficits. However, its medicinal benefits include helping to relieve nausea caused by specific cancer treatments and exerting an anti-inflammatory effect [28]. On the other hand, CBD has analgesic and neuroprotective effects and properties that relieve discomfort in people with cancer and diabetes. Differences in CBD–THC ratios delineate three types of classes: type I (ratio < 0.5), type II (ratio 0.5–3.0), and type III (ratio > 3.0) [1,14]. The concentrations of these compounds and others, such as terpene phenolics, are dependent on the age, type of tissue, variety, and environmental and growth conditions, such as nutrients, humidity, and the photoperiod of the plant [16].

#### 3.1.2. Terpenes

Terpenes can be easily extracted from the raw material by using steam distillation. They are also called essential or volatile oil. They have been used as an anti-inflammatory in the respiratory and digestive tract and have become economically significant in flavors and fragrances. Terpenes are lipophilic compounds that can pass through membranes quickly. They can alter the THC pharmacokinetics by permeating the blood–brain barrier, functioning as a permeating agent, and modulating the affinity of THC for the CB1 receptor. Therefore, they present a broad range of medical properties depending on the terpene [29]. Likewise, they are responsible for the smell and taste of different varieties of *Cannabis*. Over 120 terpenoid compounds have been identified in the plant; mono- and sesquiterpenes, with 10 and 15 carbons, respectively, are detected in the flowers, roots, and leaves. Triterpenes, with 30 carbons, have been detected in the roots and fibers of hemp, as well as in the oil of *Cannabis* seeds [16] (Table 1). It is necessary to further characterize terpenes and terpene profiles from Cannabis since robust analytical standards are lacking, and some terpene compounds are still unknown. The terpene composition in cannabis-extracted oil or resin is dependent upon genetic, environmental, and evolutionary factors, as well as differences between individual plants [29,30].

#### 3.1.3. Phenolic Compounds

The phenylpropanoid pathway in the cytoplasm produces phenolic compounds, which are subsequently transported in the vacuole or deposited on the cell wall. Although the flavonoid pathway has been extensively studied in various plants, there are no specific data on flavonoid biosynthesis in *Cannabis*. In general, in plants, phenolic compounds can act as antioxidants under certain physiological conditions, protecting plants from oxidative stress. In humans, it has been shown that there is a correlation between the intake of phenolic compounds in the diet and a lower incidence of chronic diseases such as cancer and cardiovascular and neurodegenerative diseases [31], but these positive health effects cannot be totally understood because the compounds are poorly bioavailable.

In *C. sativa*, thirty-four flavonoids have been isolated, which can be categorized into seven bare chemical skeletons: ethylated, glycosylated (C or O glycosides), prenylated, or geranylated [32]. Most of these are flavones (apigenin and luteolin), flavonols (kaempferol and quercetin), aglycones, or glycosides [33,34]. In *C. sativa,* cannflavin A, B, and C have been isolated and represent hemp-specific methylated isoprenoid flavones. It is known to possess anti-inflammatory action [35]. In Central Italy, quercetin, naringenin, and naringin were identified and quantified in a hydroalcoholic extract from hemp inflorescences of monoecious cultivars [36].

Another class of polyphenolics, dihydrostilbenoids, have been isolated from Cannabis, with canniprene being the primary representative [37]. In *C. sativa*, higher flavonoid content has been reported in the leaves than in the other plant tissues, and the concentration seems to decrease with plant tissue age. Thus, higher flavonoid content is found in young cannabis plants [33].

## 4. Current Status of *Cannabis* Extraction Techniques

The extraction methods used to obtain cannabinoids and other bioactive ingredients represent a critical and economically important step, mainly when they can be used as pharmaceutical, cosmetic, and food product ingredients. This step is not understood and needs to be reviewed. Furthermore, from the laboratory scale to the pilot or industrial scale, there are several variables to consider to select the best extraction method (purity yield), which include variety selection, cultivation, harvesting, extraction technique, plant material pre-treatment, etc. [38].

### 4.1. Current Conventional and Emerging Technologies for Cannabinoid Extraction Processing

Before extracting cannabinoids and other biocompounds from Cannabis, previous stages need to be considered. These include selecting, drying, pulverizing, and sieving the plant material [39]. Another treatment (before or after extraction) consists of decarboxylation to convert acid cannabinoids to their neutral psychoactive forms by heating at 100–150 °C for 15–120 min [40].

Traditional methodologies for extracting cannabinoids from cannabis materials utilize volatile solvents (mainly ethanol), liquid propane or butane, supercritical CO_2_ (SC-CO_2_), or ice water. Several medicinal cannabis extract procedures use alcohol as a solvent because of its high efficiency and food-grade quality. However, solvents such as butane, propane, or hexane are of limited use because they can produce an uncontrolled hazardous environment.

Some laboratory, pilot plant, and industrial extraction processes have been developed to obtain and improve the quality and purity of the main cannabinoids or terpenes, and some examples of extraction techniques are described in this section and Table 2.

Currently, the most potent type of oil extract is called Full-Extract Cannabis Oil (FECO). It has the highest concentration of cannabinoids, usually composing between 50 and 80 percent of the weight. Other less potent extracts are tinctures, which are liquid extractions made primarily with alcohol, glycerin, olive oil, or coconut [52].

#### 4.1.1. Butane Hash Oil Extraction (BHO)

**Figure 2:**
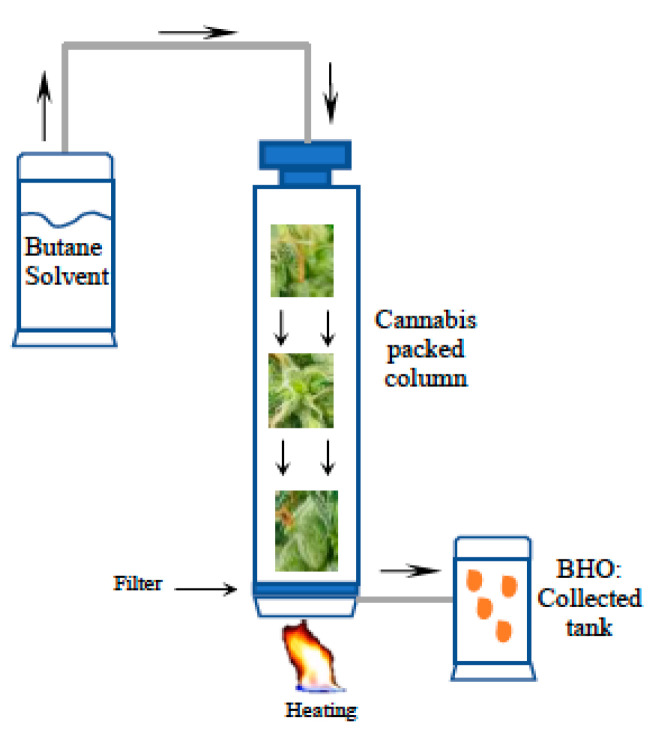
Butane Hash Oil extraction system. Cannabis in put into a column or extraction cylinder and butane solvent is passed through, heated a low temperature to extract terpenes and cannabinoids. The extract is then filtered and recovered into a new cylinder.

Using liquid butane gas to extract cannabinoids from *Cannabis* flowers has made it possible to obtain a product called butane hash oil (BHO) with a THC concentration higher than that found in the flowers [53]. Between 1993 and 2015, the THC content in seized BHO samples had maximum concentrations of up to 90% compared to the 37.1% obtained in cannabis flower samples [54]. BHO is also known as “amber”, “dab”, “glass”, “honey”, “shatter”, or “wax”. The extraction process consists of dissolving cannabinoids and terpenes from the plant material using a hydrocarbon, most commonly butane, to obtain a concentrate in which the most significant component is THC. Butane is non-polar. Thus, the extraction process cannot extract some soluble compounds, such as chlorophylls or alkaloids. Butane gas (highly flammable and volatile) permeates the air and can be ignited by a flame source (Figure 2). After extraction processing, butane is purged by evaporation to obtain BHO [55]. Like other extraction processes, decarboxylation and winterization are essential steps to obtain BHO [39]. Butane extraction can be risky if there is no closed-loop system to recover and recycle butane, a highly volatile and flammable compound that accumulates in closed spaces [55,56].

Non-commercial extraction or home use can quickly adapt BHO for extraction processing. However, it can be dangerous if extraction areas are not in optimally ventilated to eliminate butane vapor accumulation. On the other hand, the BHO obtained may contain a certain amount of butane, methacrolein, and benzene (products of the degradation of terpenes at elevated temperatures), which may cause acute lung injury and pulmonary edema [57]. Regarding patent research, when using the term “butane hash oil”, 32 patents were found in 2022 on the Scopus platform.

#### 4.1.2. Soxhlet Extraction (SE)

Soxhlet extraction is another process developed to obtain essential compounds from the cannabis plant. This method uses solvents, is rapid and efficient, and is suitable for large-scale operations. The process consists of heating the plant material under reflux, and the main solvent used is ethanol [58]. Ethanol is added to a container of Cannabis to react in heating and reflux conditions for at least one hour [59]. The resulting extract is then clarified to separate the solids from the solution saturated with cannabinoids and terpenes. The saturated ethanol solution is then boiled to concentrate the cannabinoids. For industrial capacity, this system can be adapted. However, it can be pretty costly. It is necessary to balance the technical and economic benefits that can be realized by implementing this extraction technology [58]. It is essential to mention that ethanol vapor can be recovered using various strategies or distillation steps. The extraction solvent also can be used as a first step for subsequent extraction (supercritical CO_2_, microwave heating, etc).

#### 4.1.3. Cold Press Extraction (CPE)

Hemp seeds are high-value components, with approximately 25–35% lipids, 20–25% proteins, 20–30% carbohydrates, 10–15% insoluble fibers, and numerous natural source minerals [60]. This technique has been used to extract fatty acids and bioactive compounds from cannabis seed oil. When the seed is clean and ready for extraction, it is ground in mills, cylinders, or spurs to break the seeds, and the oil comes out. These mechanical pressing extraction systems are considered cold extraction methods. Pressing is a standard method that uses low-cost and simple technology. In cold pressing, the seeds pass through a low-speed and low-pressure press, whose internal temperature can be kept below 50 °C. However, when a screw press is used, it is heated to improve the extraction yield, which can produce some pleasant sensory characteristics in the extracted oil [61].

The semi-defatted paste or oil sludge resulting from the pressing process may be required for further solvent extraction processing or processed a second time with a press with high pressure and speed that separates the oil from the remaining seed paste for the recovery of the remaining oil [62]. In general, this process has the advantage of preserving the properties of the oils, although with a lower yield.

#### 4.1.4. Supercritical CO_2_ Extraction (SC-CO_2_) 

Supercritical CO_2_ has been used to extract high-added-value compounds such as nutraceuticals and pharmaceuticals. Additionally, toxic compounds such as pesticides are removed from solid and liquid matrices [63]. Supercritical CO_2_ extraction has been used to obtain a high-ԛuality product without any toxic residue by the Cannabis industry over the last 22 years [64,65].

Extraction with *SC-*CO_2_ consists of the use of CO_2_ at a pressure and temperature higher than its critical values or critical points (31.3 °C and 72.9 atm), which causes it to present properties of a liquid and dense gas at the same time, facilitating the extraction and separation of the dissolved solute from SC-CO_2_ after decompression [66]. Supercritical CO_2_ has been used to extract hemp (*C. sativa*) seed oil, obtaining a greater quantity of tocopherols and a lower quantity of pigment than extraction by Soxhlet and cold press processing [67].

Briefly, the process includes the industrial hemp material, followed by a treatment that reduces the particle size (cutting or grinding). Then, the cut material is settled in a pressure vessel, and SC-CO_2_ is passed into the container. SC-CO_2_ is preferably maintained at 40–100 °C and 150–300 bar pressure. After that, the CO_2_-dissolved compounds are collected in a lower-pressure container and precipitate as CBD crude extract. The crude extract may contain cannabidiol acid (CBD A), and it is possible to convert CBD A to CBD by decarboxylation using heating (100–120 °C). The crude CBD extract can be subjected to a purification step.

According to Benner et al. [68], there is considerable variability in the temperature (113 to 140 °C) and pressure ranges (3000 to 5000 PSI) that different extractor facilities use for the process. These conditions can affect the quality and yield of cannabinoids, terpenes, and flavonoids. Greater temperatures increase the risk of denaturing terpenes and increase waxes and resins, which decrease the overall extract quality. It may be possible that applying pulses of ethanol (co-solvent) improves the extraction speed with low consumption of the co-solvent and CO_2_ in strains of *C. sativa* L. with a low concentration of cannabinoids [41].

The use of ethanol as a co-solvent improves the extraction process. In addition, the decarboxylation of cannabinoids (increased solubility in SC-CO_2_) and winterization (to remove waxes present in the flowers) are still crucial steps that enhance the extraction of THC and CBD [69]. Additionally, using other solvents or re-using ethanol to minimize operating costs is possible. The SC-CO_2_ process is presented in International PCT Publication WO2016/153347, which is directed at a cannabidiol isolate from industrial hemp for use in pharmaceutical or cosmetic preparations.

The first study, which was reported by Rochfort et al. [70], included an optimized CO_2_ supercritical fluid extraction (SFE) process protocol without a co-solvent for a large amount (15 kg) of medicinal cannabis bud material for a pharmaceutical product. The highest extraction (7.1%) was produced under a high flow rate (150 g/min), with a long extraction time (10 h) at high pressure (320 bar). A comparative study between the cannabinoid and terpene concentrations before and after SC-CO_2_ extraction resulted in a significant reduction in the terpenes and a significant increase in the THC and CBD contents in the final extract. 

The main advantages of SC-CO_2_ seem to be a clean, safe (non-flammable), and cost-effective alternative to obtain cannabis extract. Nowadays, many equipment suppliers can be easily identified. However, one of their disadvantages is the excessive cost of the equipment. A typical large-scale unit can cost from USD 300,000 to USD 10 million [68].

#### 4.1.5. Microwave-Assisted Extraction (MAE)

Microwaves have been used as a heating method to extract organic compounds such as polycyclic aromatic hydrocarbons, polychlorinated biphenyls, pesticides, herbicides, and phenols from several types of matrices [71,72]. Microwave-assisted extraction (MAE) consists of heating the polar-solvent–sample mixture by ionic conduction and dipole rotation mechanisms through the application of an electromagnetic field (300 MHz to 300 GHz), which reduces the processing time and the solvent volume used [73]. Microwave-assisted extraction (MAE) has been used successfully to extract compounds of interest from plants [74]. During the MAE of hemp inflorescences, an aqueous residue and the residual deterpenated plant biomass remain in the reactor as by-products, being a valuable source of flavonoids and phytocannabinoids [75].

The variation in parameters such as the solvent concentration, extraction time, and solid–liquid ratio (S/L) has been shown to influence variables such as the yield, IC_50_, EC_50_, CBD, THC, total flavonoids (TF), and total phenols (TP) from *C. sativa* [42]. In recent research work, the influence of variables on the MAE efficiency (microwave irradiation power, extraction time, and added water) was evaluated to obtain high-quality products: essential oil, an aqueous extract rich in polyphenols, and phytocannabinoids [76]. On the other hand, microwaves at 915 MHz have been established in continuous operation on an industrial scale for the extraction of cannabis compounds. It is possible to process larger volumes of matter in less time with a high extraction efficiency (>95%) [77]; despite its positive characteristics, the potential for use at an industrial scale is limited. 

#### 4.1.6. Ultrasound-Assisted Extraction (UAE) 

Ultrasound (>20 kHz) has been used to assist extraction. Bubble cavitation facilitates the extraction of polysaccharides, edible oils, essential oils, proteins, peptides, pigments, and other bioactive molecules in diverse matrices [78]. The cavitation process generates changes in temperature and pressure at a bubble level, facilitating the transfer of matter and reducing the volume of the solvent and the extraction time [79]. Ultrasound-assisted extraction (UAE) has been used to extract bioactive compounds from *C. sativa* L., and optimizing the process using 80% methanol, 15 min, and 130 W improved the extraction of THC and CBG from *C. sativa* L. [47]. In another study, the optimized extraction conditions for volatile compounds and cannabinoids were 3 s^−1^ cycles, an 80% amplitude, 5 min of sonication, and a 1:1 isopropanol/cyclohexane mixture [80]. However, the same study discusses the disadvantage of ultrasound in separating terpenes from cannabinoids compared to using SC-CO_2_ and ethanol as a co-solvent. Compared to MAE, the UAE method produces better THC and CBD extraction yields [71].

#### 4.1.7. Pressurized Liquid Extraction (PLE, ASE, HSPE)

Pressurized liquid extraction (PLE), accelerated solvent extraction (ASE), or high-pressure solvent extraction (HSPE) is considered a green solid–liquid extraction technique for sustainable bioactive compounds from natural sources. PLE is conducted by applying heat and pressure to the extraction solvent to which the sample is subjected. The extraction rate and efficiency are related to the sample solubility, solvent, rate of mass transfer, extractability of compounds, pressure, and temperature [81].

Recently, Olejar et al. [82] developed a method for the thermo-chemical conversion of acidic cannabinoids during pressurized liquid extraction to isolate cannabidiol. An optimized PLE method using water for thermo-chemical conversion before an ethanol extraction step was evaluated. The extraction process combined with purification by flash chromatography and liquid–liquid extraction yields cannabidiol crystals of greater than 90% purity. In other research work, the influence of temperature, pressure, extraction time, and the number of cycles for the PLE of cannabinoids from hemp was studied [46]. 

PLE has also been evaluated for CBD extraction from hemp threshing residue with ethanol, where the solvent mass-consumption and operation time were decreased compared to a supercritical fluid extraction technique [83]. 

All these results and conditions show that this technology could have the potential for cannabinoid extraction from various parts of the plant. 

#### 4.1.8. Deep Eutectic Solvent Extraction (DESs)

Advanced research extraction processing and applications to obtain different molecules have attracted extensive attention, with a new generation of green solvents that can be used. The deep eutectic solvent (DES) has been evaluated and was reported to have properties like those of ionic liquids. For the first time, Křížek et al. [84] evaluated the effect of hydrophobic DESs based on menthol and natural carboxylic acids on the efficiency with which they extracted phytocannabinoids (THC, CBD, THCA, and CBDA) from the cannabis plant material. The DES mixture of menthol–acetic acid (1:1 M ratio) showed excellent extraction efficiency and was a non-toxic and biodegradable alternative to organic solvent for phytocannabinoid extraction. Furthermore, these solvents have advantages such as low toxicity, low precursor cost, simple synthesis, little volatility, and high biodegradability [51].

#### 4.1.9. Emerging Extraction Technologies

For cannabis extraction, other extraction techniques can be considered, such as extraction, enzyme-assisted extraction (EAE), microwave distillation (MD), pulsed electric field (PEF), emerging subcritical-butane-based, and emerging solvent-free extraction techniques.

For example, an emerging solvent-free extraction, namely, a new solvent-free method for extracting the full spectrum of phytocannabinoids, terpenes, and flavonoids from the whole cannabis/hemp plant, was patented by [68]. Furthermore, n-butane has been a more than valid alternative to n-hexane and petroleum ether for the extraction of lipophilic natural products. In the case of Cannabis, this method also has wide use [85]. The subcritical-butane-based extraction solvent for cannabinoids from hemp inflorescences was reported for the first time by Fiorito et al. [86]. The pulsed electric field (PEF), a nonthermal technology, has been applied to extract oil from *Cannabis* seeds with promising results. Higher extraction efficiency improved the oil quality with less thermal degradation [87,88] Combining two or more technologies can be considered to obtain a better Cannabis or bioactive compound extract.

Higher bioactive compound or cannabinoid yields can also be obtained using emerging extraction techniques combined with fast and efficient separation. Kitrytė et al. [89] evaluated a multistep biorefining technology for isolating valuable phytocannabinoids and antioxidant fractions from industrial hemp threshing residues via the consecutive application of SFE-CO_2_, PLE, and EAE.

Although these emerging technologies (EAE, PEF, MD, and SFE-CO_2_) are expensive, they could be excellent options for considering the potential and effective extraction results of phytochemicals from *Cannabis*.

### 4.2. Purification Step 

Some products can use the oil or aqueous extract to obtain products such as cannabis oils, vaporization substances, emulsions, food additives, edibles, drinks, and oral sprays. For example, medical or therapeutic products require beyond 99% purity.

Thus, once the extraction process has been carried out, the extract may contain non-specific lipid-soluble materials or waxes, which can be removed by a traditional purification process such as winterization. Winterization consists of the ethanol solubilization of the resin, followed by precipitation at 20 °C and wax filtration. Subsequently, ethanol and other residual solvents can be removed by evaporation at 40–60 °C [39].

Depending on the desired final product, the extracts can be subjected to additional purification steps, such as vacuum distillation or short-path distillation (molecular distillation) to produce a pure cannabinoid (90%) [90] or centrifuge chromatography to isolate cannabinoids with high-purity content (99%) [90,91].

For example, Cannabis oil can be further fractionated into distillate and a residue by molecular distillation, also known as “short-path” distillation. Molecular distillation is used to separate and purify heat-sensitive compounds and high-molecular-weight compounds and is identified as a “safe method” [92,93,94].

**Figure 3:**
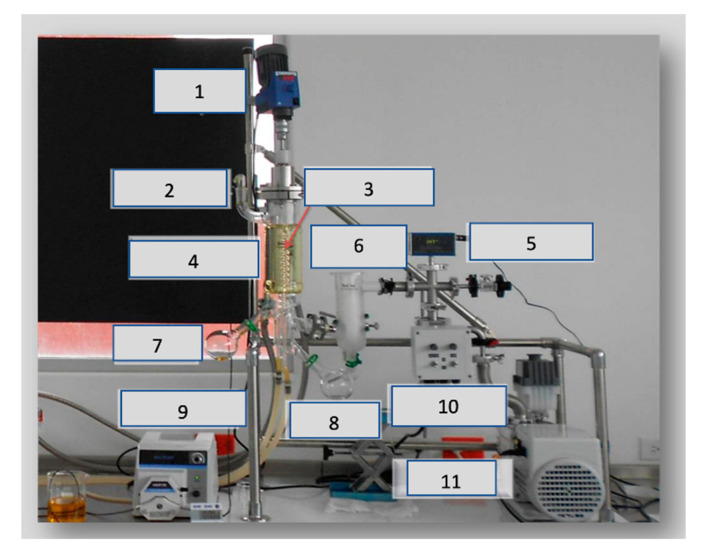
Lab-scale InCon ICL-04A short-path distillation. 1. Mixer; 2. feeding; 3. condenser; 4. evaporator; 5. vacuum gauge; 6. vacuum trap; 7. residue; 8. distilled; 9. peristaltic pump; 10. pump; 11. vacuum pump.

This method is based on high vacuum pressure (1–0.001 mbar), which allows the molecules that escape from the evaporation surface to reach the surface and condense before colliding with each other [95,96]. The vacuum condition allows a short residence time and a small holdup volume [97]. According to the equipment used, molecular distillation can be of the centrifugal type or the falling film type, depending on whether the liquid to be distilled is distributed as a thin film on the evaporation surface through a laminar flow or a centrifugal force, respectively [98,99] (Figure 3). Molecular distillation has also been part of post-extraction refinement processes to obtain distillate cannabinoids (90%) [91,100,101]. The CBD crude extract can be subjected to thin-film evaporation to produce a refined extract. Molecular distillation is preferably performed at a temperature within 100–160 °C. 

Thus, different technological and experimental design strategies can be used for cannabinoid extraction and production. It is essential to note that the final application and the compound of interest define the most efficient process to obtain the desired product [102].

## 5. Current Research on Extraction Processing in Latin America 

Worldwide, the industrial and scientific interest in *Cannabis* has been increasing. Therefore, rapid and accurate methodologies for the quality extraction of the main components will be crucial. Cannabinoid extraction can be associated with compound–matrix interactions, which depend on the variety, harvesting, environmental factors, and selected extraction technique. The extraction efficiency is also influenced by operational parameters, such as the temperature, time, solvent, physicochemical properties, extraction technique, and *Cannabis* strains. 

To date, *Cannabi*s and cannabis formulations of several varieties of medical marijuana concentrate, including edibles, are being marketed without testing regulations. As Cannabis is a natural product that can be susceptible to pests and microbial deterioration (bacterial and fungal attack), careful extraction and quality control must exclude these and other potentially dangerous contaminants from medicinal products [103]. 

The leading research of extraction technologies for *Cannabis* has been widely performed in Europe and the USA. However, there is interest in these technologies and commercialization opportunities in Latin America, especially in Brazil, Uruguay, Chile, Colombia, and Mexico, where cannabis legalization requests are in progress to eliminate illegal trafficking while obtaining beneficial health impacts.

**Figure 4:**
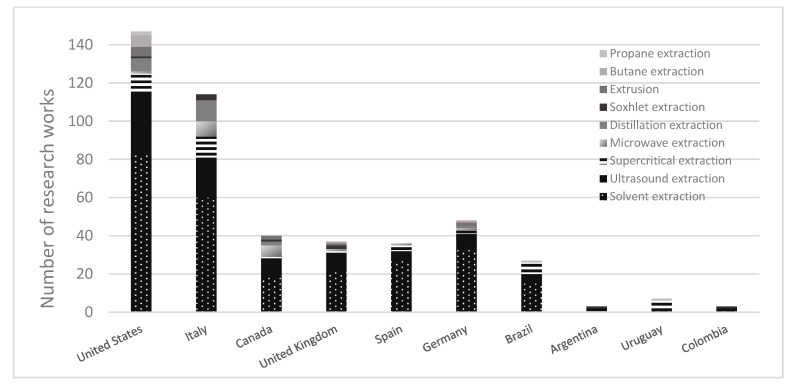
Worldwide publications of cannabinoid extraction technologies.

It is evident that there is a need for more original research and designs of extraction technologies and equipment from developing countries. However, in the last few years, the increase in publications has been undeniable, influenced by research and development in Europe and North America. Several publications from the Scopus database (2000–2022) are shown in Figure 4. The worldwide research demonstrates that the leading cannabis extraction technology studied is solvent extraction, followed by ultrasound-assisted extraction (UAE) and supercritical extraction (SC-CO_2_). When these results are compared to the primary extraction techniques in Latina America, solvents and supercritical extraction are the most studied in these countries. 

In Brazil, legislation indicates that growing *Cannabis* is still forbidden. It is only permitted with legal authorization. However, developing technologies to obtain cannabinoid extracts with health benefits represents an active area of research. Brazilian research includes extraction technologies evaluated in various plant parts to obtain cannabinoids, fatty acids, and terpenes. Some of them have used pressurized n-propane as a solvent for the extraction of hemp oils as an alternative to improve extraction efficiency without changing their nutritional qualities. n-Propane demonstrated improved oil solubility and extraction. When the studies are aimed at producing, characterizing, and quantifying extracts enriched in cannabinoids, solvent extraction by using maceration (methanol, diethyl ether) is commonly used. When essential oils and cannabinoids (CBD, Δ^9^-THC, and CBN) are the target compounds, pure SC-CO_2_ with ethanol as a co-solvent has been preferred as an extraction technique (see Table 3).

In research works developed in Colombia, cannabidiol was extracted at up to 66% from the original biomass using a semi-continuous lixiviation process with absolute ethanol as the solvent and a constant temperature and stirring speed. This study provided a more efficient extraction method compared to Soxhlet extraction (efficiency of 10.5% vs. 11.07%, respectively) and with a meager initial investment compared to other techniques, such as SC-CO_2_ [104] In 2022, Vega and Dávila [105] studied the extraction time, particle size, and solid–solvent ratio using Soxhlet extraction (ethanol as a solvent) to extract phenolic compounds from the leaves and stems of *Cannabis sativa* L. These parts of the plant are considered residual biomass with a reliable source of bioactive compound extracts and attractive economic and environmental advantages. Similar to Brazilian research, maceration (using ethanol) and SC-CO_2_ extraction for comparison were evaluated and reported as two of the most used methods to obtain cannabinoid extracts for medical purposes. The SC-CO_2_ extraction conditions led to the highest total THC and CBD recovery [106].

The pharmacological and medicinal potential of cannabinoids has been a topic of interest in Uruguay and Brazilian universities. To demonstrate this effect on various human tumor cells, extracts of *Cannabis* flowers were obtained by SC-CO_2_ (ethanol as a co-solvent) at low temperatures and without organic solvents using a previous decarboxylation step. Using this extraction technique at 40 MPa and 50 °C resulted in a high yield and high Δ^9^-THC content. The extracts with high concentrations of neutral cannabinoids showed high antitumor activity in cervical cancer cell lines [69].

The first country in the world to regulate the production of *Cannabis* for recreational, medicinal, and industrial use was Uruguay. In Uruguay, CBD is the main export product for medical *Cannabis*. Then, the market will demand significant amounts of CBD in the next few years. Thus, a regulatory point of view concerning the health, medicinal, and nutritional properties of industrial cannabis products has been necessary. In 2021, Uruguayan research concluded that it is crucial to consider the standardization of chemical profiles of *Cannabis sativa* extracts used in medicinal cannabis oil. These profiles could be modified due to different extraction and purification conditions, such as the temperature (solvent evaporation process) or chemical reactions due to oxygen. These chemical changes must be carefully considered in the development of medicinal oils.

Argentina and Mexico are waiting for new regulations to generate new horizons to initiate investigations of medicinal *Cannabis* products that demonstrate safety and evidence in treating different pathologies, carry out studies in experimental models, advance the genetics of the plant, and consolidate production. In agreement with the extraction techniques used in Latin America, *Cannabis sativa L.* extracts with a high concentration of Δ^9^-tetrahydrocannabinolic acid (THCA) and Δ^9^-tetrahydrocannabinol (THC) were obtained by supercritical carbon dioxide (SC-CO_2_) extraction in Argentina. The extraction yield was highly dependent on the pressure and raw material quality composition, and a process extraction efficiency as high as 92% was achieved [41].

In México, in the same situation as Brazil, public research is not permitted without legal authorization. Thus, only a few review papers were identified. One of them reviewed state-of-the-art *Cannabis* patents and included specialized/secondary metabolite isolation methods, synthesis, production, genes, development of medicaments, drug delivery systems, equipment, machines, industrial processes, plant culture techniques, and improved plant varieties [107]. In another review, the term “Tetrahydroevolution of cannabis” was suggested by the author, where this cannabis tetrahydroevolution can be divided into three main dimensions: (1) the neo-production of Cannabis, (2) the neo-refining of raw materials, and (3) the neo-presentation of products derived from cannabis [108]. These dimensions have been referred to for the recent superior cannabis development reported in the last several decades compared to its preceding quality. A third paper reviewed and discussed the latest development works aiming at the innovative extraction of cannabinoids and purification of CBD using traditional, emerging, and synergistic extraction techniques and strategies and their relevant outcomes [109] (Table 3).

**Figure 5:**
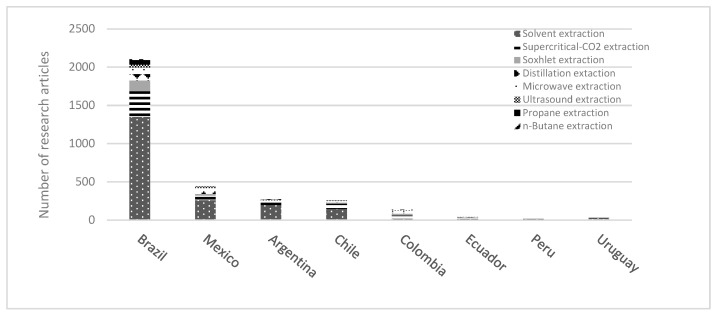
Extraction processing techniques in research articles contributed by Latin American countries.

This analysis shows opportunities for *Cannabis* in Latin American research and development. Data from the Scopus database highlight the extraction methods used by Latin American countries over the last ten years of research. These techniques have been used to optimize the conditions to obtain metabolites from other plant materials, such as corn, seeds, oil extract, nut shells, marine microalgae, etc. [110,111,112,113,114] (Figure 5). This information is critical to appreciating the technological potential to implement these methodologies for cannabis research. However, extraction technologies tailored to *Cannabis* in some Latin American countries have been discontinued by their legislation.

**Table 3:** Contribution to research on cannabis extraction technologies by Latin American countries.

Country	Biomass Form	Target Compound	Extraction Technology	Conditions	References
Brazil	Seeds	Fatty acids Tocopherols β−Carotene	Pressurized n-propane	Temperature: 40, 50, and 60 °C Pressure: 6, 8, and 10 MPa	[115]
	General biomass: forensic samples	THC/CBD	Solvent extraction: Dynamic maceration methanol, and diethyl ether	Heating	[116]
	Hybrid flowers (2 varieties)	Cannabinoids	Green solvent extraction: supercritical carbon dioxide (SC-CO_2_)	Temperature: 50, 60, 70 60 °C Pressure: 200, 300 bar Co-solvent: 0, 10% EtOH	[50]
	Standard	CBD	Supercritical carbon dioxide (SC-CO_2_)	Temperature: 315, 326, 334 K Pressure: 11.3–19.4 MPa	[117]
Colombia	Cannabis with fully ripe inflorescence	THC	Supercritical fluid extraction (SFE) using CO_2_–ethanol	Pressure: 15–33 MPa Temperature: 40–80 °C Co-solvent: (0–5%) EtOH	[118]
	General biomass (flowers, leaves, stems, and other parts)	Cannabinoids (CBD, CBDA, CBC, CBG, THC)	Soxhlet extraction compared to semi-continuous lixiviation process	Soxhlet extraction: 2 h, solvent/biomass ratio of 6:1 Single-stage extractions: ethanol (40 g and 2 g of biomass) Temperature: 40 and 19 °C	[104]
	Stems and leaves	Phenolic-rich extracts	Ethanol solvent extraction	EtOH 96%	[105]
Uruguay	Female inflorescences	THC/CBD Cannabinoids	Solvent extraction (short maceration) and supercritical fluid extraction (SC-CO_2_)	Temperature: 60, 70 °C Pressure: 200, 300 bar Co-solvent: 0, 10% EtOH	[106]
	Flowers	CBD/THC	Green solvent extraction: supercritical carbon dioxide (SC-CO_2_)	Temperature: 50 and 70 °C Pressure: 22 and 40 MPa	[69]
Argentina	*Cannabis sativa* extracts	Terpenoids, CBD/THC	Soxhlet and maceration extraction	Temperature: 40 and 70 °C to dryness under reduced pressure	[119]
	*Cannabis sativa* extracts	THCA, THC	Supercritical carbon dioxide (SC-CO_2_)	Pressure: 17, 24, and 34 MPa Temperature: 328 K	[41]
Mexico				Reviews	[107,109]

## 6. Future of Commercialization and Perspective on the Value-Added Chain in Latin America 

A potential niche for a boost in the cannabis derivative market is the biomedical area. Before its current illicit substance status, *Cannabis* had been used for hundred years for medical purposes. In the US, *Cannabis* was described in the *United States Pharmacopoeia* for the first time in 1850 and then widely used as a patent drug during the 19th and early 20th centuries [120].

The global medical cannabis market increased in value from USD 8.28 billion in 2017 to approximately USD 9 billion in 2020, with expected values of USD 28.07 billion in 2024, and it is expected to reach nearly USD 50 billion by 2028 [121,122]. According to another report, *The Road Map to a $57 Billion Worldwide Market*, legal cannabis worldwide is expected to hit USD 57 billion by 2027. The same report indicated that Germany is ready to become the leader of the European cannabis market, followed by Italy, with USD 1.2 billion in sales by 2027. In South America, the medical cannabis market may grow from USD 125 million in 2018 to USD 776 million in 2027, led by Brazil, Argentina, Peru, and Uruguay [123].

According to this information, the cannabis market forecast is economically strong. Nevertheless, there are still obstacles in the development of medicinal *Cannabis*. For example, the United States, Canada, and the United Kingdom designated USD 1.56 billion between 2000 and 2018 for research funding. Half of the budget was spent on understanding the potential harms of the recreational drug, approximately 20-fold the amount paid for cannabis research and development in the therapeutic area [124].

Considering health problems, cannabis oil, which is rich in medical properties, is booming, as the demand from patients with chronic illnesses, such as Parkinson’s disease, cancer, Alzheimer’s disease, and other neurological disorders, is increasing worldwide [125]. However, not all countries permit their commercialization. Thus, an increase in illegal products marketed as products for medical use or therapeutic purposes is found in these countries, especially in the Latin American market.

According to research and development, demand in the current value-added chain is mandatory. The existing value chain only has permitted crops (spread, flowering, and harvesting), distribution in legal markets, and finally, sale in local markets.

**Figure 6:**
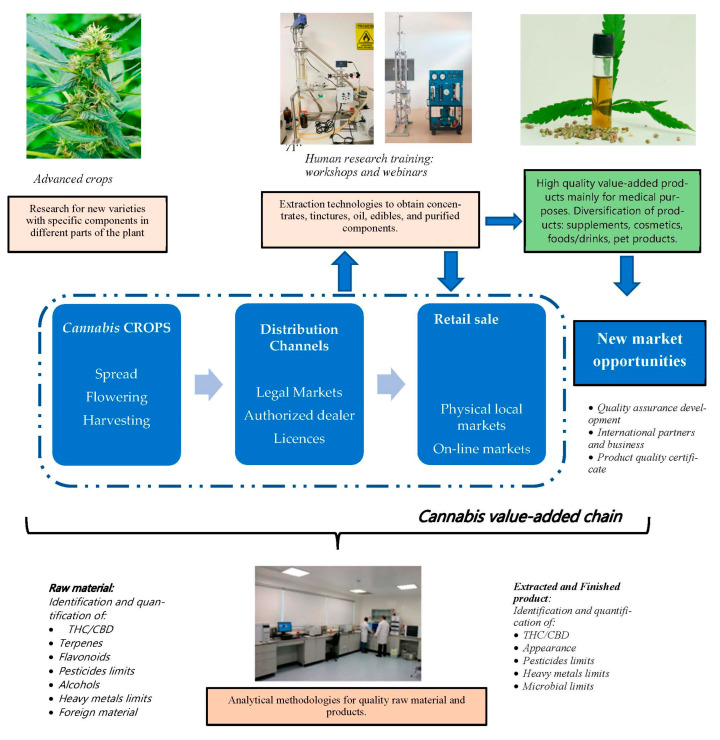
Perspectives on *Cannabis* value-added chain in Latin America include research on extractions techniques for cannabinoids to obtain diversification of high-quality products.

In Latin America, however, the perspective on the value-added chain for cannabis must include advanced crops, an official distribution channel (considering a legal market), retail sales (local and online markets), and research and development (human resource capabilities, extraction equipment, physical infrastructure) to obtain high-quality products for medical, food, or cosmetic purposes (Figure 6).

Analytical and technological tools are also necessary for industrial manufacturers to offer a safe product, improve quality, and extend product lines to diversify and open new market segments to find a more successful business model in Latin American countries.

## 7. Conclusions and Future Perspectives

*Cannabis* compounds are essential in pharmaceuticals for the treatment of depression, anxiety, insomnia, epilepsy, and seizures or as ingredients in healthy functional foods (oil, tinctures, cookies, ice cream, chocolates, butter, brownies, cooking oil, jellies, beverages, capsules, pills, etc.). In general, research and development, especially on the cannabis extraction topic, is increasing due to these positive health benefits and the local economic impact of cannabis products.

In addition, obtaining higher-quality cannabis products represents higher prices. The correct selection of the extraction and separation processes is essential, with this step being one of the biggest challenges. Latin American countries exhibit profound knowledge and availability of extraction technologies for cannabis extraction. In this sense, it is necessary to estimate the production cost to evaluate whether they are economically profitable, at least at the laboratory scale, and to obtain a final desirable product.

In Latin America, there is also an increase in commercial cannabis extract or cannabis products that may or me not be allowed, with or without quality fact sheets, which can pose health risks. Hence, legalization is mandatory and urgent for the rest of Latin American countries.

This action will allow the innovation, research, and development of cannabis products according to the value-added chain (from the raw material to the final product) expected for Latin American countries. Furthermore, it will allow the evaluation of more selective, efficient, and effective extraction and processing technologies to obtain high-quality cannabis extracts and to enable the development of new formulations with added value for medical purposes.

## Figures and Tables

**Table 1:** Terpenes found in *Cannabis sativa*. Classification according to the number of carbons, the part of the plant where they are located, and some of their pharmacological effects.

Classification	Number of Carbons	Name	Part of the Plant *Cannabi*s	Other Plants in Which It Is Found	Pharmacological Effects
Monoterpenes	10	D-Limonene	Cannabis flowers, roots, and leaves	Citrus	Anticancer, anxiolytic, and immunostimulatory
		β-Myrcene		*Humulus lupulus*	Anti-inflammatory, analgesic, and anxiolytic
		α- and β-Pinene			Acetylcholinesteral inhibitor; helps counteract THC-induced memory deficits
		Terpinolene			
		Linalool		*Lavandula angustifolia*	Analgesic, anxiolytic, anti-inflammatory, and anticonvulsant
Sesquiterpenes	15	β-Caryophyllene		*Piper nigrum*	Anti-inflammatory and gastric cytoprotective
		α-Humulene			
Triterpenes	30	Friedelin	Hemp roots		
		Epifriedelanol			
		β-Amyrin	Hemp fibers		Antibacterial, antifungal, anticancer, and anti-inflammatory
		Cycloartenol	Hemp seed oil		
		β-Amyrin			
		Dammaradienol			

**Table 2:** Cannabis plant: examples of different technical extraction processing methods, equipment, and efficiency.

Plant Section	Technology	Equipment	Conditions	Scale	Recovery	References
Leaves and buds from different strains of *Cannabis sativa* L.	Supercritical fluid extraction (SFE) with CO_2._	Waters Co. Bio-botanical extraction system	Pressure: 34 MPa Temperature: 328 K Co-solvent: ethanol Flow rate: 200 g/min	Pilot plant scale	THC: 69.41%	[41]
*Cannabis sativa* L. cv. Helena: leaves, blossoms, small structural parts of the inflorescence, and bracts were dried and ground	Microwave-assisted extraction	Domestic microwave oven (NN-E201W, Panasonic).	Irradiation power: 580 W Extraction conditions: ethanol at a concentration of 47%, time of 10 min, and solid/liquid ratio of 5	Laboratory	CBD: 1.1504 (mg/mL) THC: 0.0474 (mg/mL)	[42]
Leaves from different strains of cannabis plants grown in different climatic regions	Supercritical fluid extraction (SFE)	SFE system (Applied Separations Speed SFE-2) equipped with a co-solvent pump (Applied Separation Series 1400) and a compressor (Atlas Copco GX-4FF)	Pressure: 33 MPa Temperature: 40 °C Co-solvent: 2.0 wt% ethanol CO_2_ supercritical flow: 10 g/min	Laboratory	Papatya strain (90.82% THC, 3.71 CBD) Elnur strain (58.22% THC, 3.29% CBD) Narli strain (7.70% CBD)	[43]
Flower from *Cannabis sativa*	Supercritical carbon dioxide (SC-CO_2_)	Helix unit (Applied Separations)	Pressure: 250 bar Temperature: 47 °C Sc-CO_2_ density: 818 kg/m^3^ Co-solvent: ethanol (5% *v/w*)	Laboratory	CBD: 19.05% *w/v* THC: 13.73% *w/v*	[44]
Industrial hemp from *Cannabis sativa* L.	Combined ionic supercritical carbon dioxide-based dynamic extraction	scCO_2_ device (Jasco Corporation, Tokyo, Japan), back-pressure regulator (BP-2080, Jasco Corporation), gas/liquid separator (HC-2086-01, Jasco Corporation)	Pressure: 20 MPa Temperature: 70 °C for 2 h CO_2_ flow rate: 5 mL/min Static extraction: 30 min Dynamic extraction: 120 min. 15 min at 70 °C pre-treatment of hemp with [C2mim][OAc] and [Ch][OAc], dilution of ionic liquid (IL) with water (1:3)	Laboratory	CBD: 15.4 (mg/g) THC: 0.498 (mg/g) CBG: 0.452 (mg/g)	[45]
Hemp threshing residues from *Cannabis sativa* L.: KC Virtus and Finola varieties	Pressurized liquid extraction (PLE)	Büchi Speed Extractor E-196	Pressure: 50 bar Number of cycles: 1 Temperature: 100 °C Time: 60 min	Laboratory	CBD: 19.8 mg/g (99.3%)	[46]
Hemp from *Cannabis sativa* L.	Ultrasound-assisted extraction	Tesla 150 WS ultrasonicator fitted with 18 mm diameter titanium probe at 20 kHz	Solid–liquid ratio: 1:20 Solvent: 20% methanol Time: 15 min Frequency: 20 kHz Power output: 90 W	Laboratory	Not specified	[47]
Hemp from *Cannabis sativa* L.	A hard-cap espresso machine	Nespresso Essenza Manual XN2003 Krups	Solid–liquid ratio: 1:500 Solvent: 2-propanol Time: 40 s	Laboratory	Different results: Buds: THC, 16–95 mg/g; CBD, 0.15–0.24 mg/g; CBN, 4.3–21.0 mg/g Leaves and stems: THC, 0.87–7.2 mg/g; CBD, <0.076 mg/g; CBN, 0.9–9.7 mg/g	[48]
Dried inflorescences from *Cannabis sativa* L. hemp cultivar	Supercritical carbon dioxide extraction	Pilot unit SFT110XW System (Supercritical Fluid Technologies, Inc., Newark, NJ, USA)	Pressure: 380 bar Vessel temperature: 60 °C Restrictor temperature: 80 °C Solvent: CO_2_ Process: eight cycles of 10 min (maceration time) in static conditions and 10 min in dynamic conditions. Flow rate: 0.28 SCMH	Pilot plant scale	Extraction yield 13%. CBD 6.21 % *w/w* of dry mass; CBD 50.2 % *w/w* of extract	[49]
*Cannabis* Hybrid Flowers: “Girl Scout Cookies” and “Durga Mata II”	Supercritical carbon dioxide extraction	CO_2_ cylinder (Air Liquide Brasil Ltda., São Paulo, Brazil, 95% purity), two syringe pumps (Teledyne Isco, Lincoln, Nebraska, Model 500D), two thermostatic baths (Quimis, Model Q214M2, and Tecnal, ModelTE-184), and one extractor with an internal volume of 170 mL	Pressure: 12.8–24.9 MPa Temperature: 50–70 °C Flow rate: 25 mL/min SCMH	Bench scale unit	Extraction yield 30%. THC 77–88%	[50]
Industrial hemp *Cannabis sativa* L.	Ultrasonic-assisted extraction (UAE) with deep eutectic solvents (DESs)	KQ-5200DE ultrasonic cleaner (Kunshan Ultrasound Co., Ltd., Kunshan, China)	Solid–liquid ratio: 1:24 Solvent: 68% Choline chloride L (+)-Diethyl L-tartrate Time: 55 min Temperature: 48 °C	Laboratory	CBD 12.22 mg/g	[51]

## Data Availability

Not applicable.

## Abbreviations

ECS, endocannabinoid system; CNS, central nervous system; CB1R, cannabinoid receptor 1; CB2R, cannabinoid receptor 2; eCB, endogenous cannabinoids; ANA, anandamide; 2-AG, 2-arachidonoylglycerol; CB1, CB2, cannabinoid receptors; WADA, World Anti-Doping Agency; FDA, Food and Drug Administration; OLA, olivetolic acid; MEP, methylerythritol phosphate pathway; GPP, geranyl diphosphate; THC, tetrahydrocannabinol; THCA, Δ^9^-tetrahydrocannabinolic acid; CBD, cannabidiol; Δ^9^-THC, Δ^9^-tetrahydrocannabinol; Δ^8^-THC, (-)-Δ^8^-trans-tetrahydrocannabinol; CBG, cannabigerol; CBC, cannabichromene; CBND, cannabinodiol; CBE, cannabielsoin, CBL, cannabicyclol; CBN, cannabinol; CBT, cannabitriol; CBD A, cannabidiol acid; SC-CO^2^, supercritical CO^2^; FECO, Full-Extract Cannabis Oil; BHO, butane hash oil; CPE, cold press extraction, SFE, supercritical fluid extraction; MAE, microwave-assisted extraction; S/L, solid–liquid ratio; TF, total flavonoids; TP, total phenols; UAE, ultrasound-assisted extraction; PLE, pressurized liquid extraction; ASE, accelerated solvent extraction; HSPE, high-pressure solvent extraction; DESs, deep eutectic solvent extraction; EAE, enzyme-assisted extraction; PEF, pulsed electric field; MD, microwave distillation.
